# A Higher Estradiol Rise After Dual Trigger in Progestin-Primed Ovarian Stimulation Is Associated With a Lower Oocyte and Mature Oocyte Yield in Normal Responders

**DOI:** 10.3389/fendo.2019.00696

**Published:** 2019-10-09

**Authors:** Jialyu Huang, Xuefeng Lu, Jiaying Lin, Ningling Wang, Qifeng Lyu, Hongyuan Gao, Renfei Cai, Yanping Kuang

**Affiliations:** Department of Assisted Reproduction, Shanghai Ninth People's Hospital, Shanghai Jiao Tong University School of Medicine, Shanghai, China

**Keywords:** estradiol, oocyte yield, mature oocyte yield, dual trigger, normal responder

## Abstract

**Background:** Prior studies have shown that patients with a >10% estradiol (E_2_) rise after trigger had more oocytes retrieved than plateauing or decreasing E_2_ responders. However, multiple follicles develop at different stages of maturation during controlled ovarian stimulation (COS) and may exhibit different responses to trigger. The association between the magnitude of E_2_ increase and oocyte retrieval outcomes is still unclear.

**Methods:** This was a retrospective cohort study of 2,898 women undergoing their first COS cycles with normal response from January 2014 to December 2017 at a tertiary-care academic medical center. Patients were categorized into five groups according to the percentage increase in E_2_ levels before and after dual trigger: <10.0%, 10.0–19.9%, 20.0–29.9%, 30.0–39.9%, and ≥40.0%. Univariable and multivariable linear regression analysis were performed to explore the association between E_2_ increase and oocyte/mature oocyte yield, while logistic regression was used to assess its effect on low oocyte/mature oocyte yield (<10th percentile).

**Results:** The post-trigger E_2_ increase was negatively associated with both oocyte yield (*P*-trend < 0.001, adjusted *P*-trend = 0.033) and mature oocyte yield (*P*-trend < 0.001, adjusted *P*-trend = 0.002). Compared with a <10.0% E_2_ increase after trigger, patients with a ≥40.0% rise had fewer mature oocyte yield [adjusted mean absolute difference [MD] = −5.2, 95% confidence interval [CI]: −8.2–−1.8] and higher risk of low mature oocyte yield (adjusted odds ratio [OR] = 1.64, 95% CI: 1.04–2.60), whereas no statistical significance was found in oocyte yield (adjusted MD = −2.7, 95% CI: −6.1–0.8) and low oocyte yield (adjusted OR = 1.48, 95% CI: 0.96–2.28). In addition, the rates of implantation, positive pregnancy test, clinical pregnancy, ongoing pregnancy, pregnancy loss, and live birth were comparable among the 1,942 frozen embryo transfer cycles with embryos originating from different groups of E_2_ increase (all *P* > 0.05).

**Conclusions:** A higher E_2_ rise after dual trigger is independently associated with a lower oocyte and mature oocyte yield in normal responders. Further studies are needed to explore the efficacy of individualized time interval from trigger to oocyte retrieval based on the magnitude of E_2_ increase after trigger.

## Introduction

*In vitro* fertilization (IVF) treatment stimulates many of the physiological processes occurring in the natural menstrual cycle in a supraphysiological manner. Following follicular growth and development, oocyte maturation is initiated by provision of luteinizing hormone (LH)-like exposure, during which the oocyte transits from the metaphase I to the metaphase II (MII) stage though meiosis and attains competence for fertilization (1). Oocyte retrieval is then scheduled at a precise interval to achieve an optimal oocyte and mature oocyte yield without preovulation.

The mode by which oocyte maturation is induced has significant impact on the efficacy of oocyte retrieval, the chance of pregnancy, and the safety of IVF treatment (2). Human chorionic gonadotropin (hCG), as the most commonly used agent to trigger final oocyte maturation, shares structural similarity, and activates the same receptor as LH (3). However, owing to the prolonged half-life and the sustained luteotropic activity of hCG (4), the risk of ovarian hyperstimulation syndrome (OHSS) is increased in patients hyperresponsive to ovarian stimulation. As an alternative to hCG, gonadotropin-releasing hormone agonist (GnRHa) elicits an endogenous surge of LH and follicular stimulating hormone (FSH) by acting at the GnRH receptors in the pituitary gland, leading to the reduction of OHSS incidence, and the retrieval of more MII oocytes (5, 6). To rescue the defective luteal phase function and subsequently comprised pregnancy outcomes caused by the GnRHa-induced luteolysis (6), the new concept of dual trigger has been introduced that combines a single bolus of GnRHa with a small dose of hCG (7, 8).

During the natural menstrual cycles, serum estradiol (E_2_) levels increase in parallel with folliculogenesis and typically decrease after the initial LH surge (9). In contrast, the LH surge that could result in premature ovulation is blocked in IVF cycles through the pituitary desensitization with GnRHa, administration of GnRH antagonist, and more recently, endogenous or exogenous progesterone use (10–12). Timing of trigger is decided mainly based on the size of follicles to mimic the midcycle surge of LH activity. While close monitoring of serum E_2_ level constitutes a vital component in assessing ovarian response to controlled ovarian stimulation (COS), limited studies have focused on the prognostic value of E_2_ change before and after trigger (13–18). Despite the controversy on whether estradiol response associates with pregnancy outcomes in IVF cycles followed by fresh embryo transfer, a consensus seems to be reached that patients with a post-trigger E_2_ rise had more oocytes retrieved than plateauing or decreasing E_2_ responders (13–18). Nevertheless, given that multiple follicles develop at different stages of maturation during COS and may therefore exhibit different responses to trigger (2, 19, 20), we hypothesized that a higher degree of E_2_ rise might be reflective of a more asynchronized follicular development, consequently contributing to a lower yield of oocytes, and mature oocytes.

The aim of the present study was to investigate the relationship between the magnitude of E_2_ increase after dual trigger and oocyte and mature oocyte yield among normal responders.

## Materials and Methods

### Study Design and Participants

This was a retrospective cohort study performed at the Department of Assisted Reproduction of Shanghai Ninth People's Hospital affiliated with Shanghai Jiao Tong University School of Medicine. The study protocol was approved by the hospital's Ethics Committee (Institutional Review Board) (No: 2017-17). Infertile women aged 21–45 years who underwent their first IVF/intracytoplasmic sperm injection (ICSI) cycle were enrolled from January 2014 to December 2017. Analysis was limited only to normal ovarian responders who had an E_2_ increase after trigger. Normal ovarian response was defined as an E_2_ level of 500–4,000 pg/mL on the day of triggering and the retrieval of 4–19 oocytes (21). Patients were excluded from the study if they met one of the following criteria: (1) use of hormonal contraceptives for pretreatment before the study cycle; (2) E_2_ concentration reaching the upper limit of our laboratory assay on the day after trigger; (3) suboptimal LH response, defined as a serum LH level of ≤ 15.0 IU/L after dual trigger (22, 23); (4) determined genetic mutation that cause arrest in oocyte maturation, fertilization, or early embryonic development (i.e., *TUBB8, PADI6*, and *WEE2*) (24–26); (5) documented history of ovarian surgery (i.e., laparoscopic ovarian drilling, ovarian endometrioma stripping and unilateral oophorectomy); (6) previous diagnosis of congenital uterine abnormality (i.e., uterus unicornis, septate uterus, duplex uterus, and uterus bicomis); (7) history of recurrent spontaneous abortion, defined as three or more previous spontaneous pregnancy losses; (8) abnormal chromosome karyotype in either of the partners; (9) core data missing in the medical records (i.e., E_2_ level on trigger day). Polycystic ovarian syndrome (PCOS) was identified according to the modified Rotterdam diagnostic criteria (27), while diminished ovarian reserve (DOR) was defined by either a serum day 3 FSH value ≥11.4 IU/L or a total antral follicle count (AFC) ≤ 4 (28).

### Ovarian Stimulation Protocol

A novel COS protocol named progestin-primed ovarian stimulation (PPOS) was employed in the present study (11, 12). Based on the freeze-all strategy, this protocol takes advantage of progesterone (P) for preventing premature LH surges as an oral alternative to GnRH analogs. Previous studies have demonstrated its efficacy in comparison with conventional short protocol (11), and proved its safety in followed-up IVF newborns regarding neonatal outcome and congenital malformations (29).

From menstrual cycle day 3 onward, patients were administered daily with 150–225 IU human menopausal gonadotropin (hMG; Anhui Fengyuan Pharmaceutical Co., China) and 10 mg medroxyprogesterone acetate (MPA; Shanghai Xinyi Pharmaceutical Co., China). The hMG dose initiated at 150 IU per day for patients with an AFC >20 or a slightly elevated basal FSH of 7–10 IU/L, while 225 IU was used for all other patients. After 5 days of stimulation, the dose was adjusted on the basis of ovarian response, as assessed by transvaginal ultrasound examination, and serum E_2_ concentration every 2 or 3 days. When at least one dominant follicle reached 18 mm in diameter, final oocyte maturation was co-triggered using 0.1 mg triptorelin (Decapeptyl, Ferring Pharmaceuticals, Germany) and 1000 IU human chorionic gonadotropin (hCG; Lizhu Pharmaceutical Trading Co., China). Transvaginal ultrasound-guided oocyte retrieval was scheduled at 36–38 h after trigger. All follicles with diameters over 10 mm were retrieved.

### Micromanipulation and Embryo Culture

The aspirated oocytes were fertilized by conventional IVF and/or ICSI according to semen parameters. For ICSI cycles, oocyte maturity status was examined 2 h after oocyte retrieval by cumulus stripping. ICSI was performed for MII oocytes at least 1 h after the removal of cumulus cells, while the other oocytes were discarded. In cases of IVF cycles, the cumulus-oocyte-complexes (COC) were inseminated first with about 300,000 progressively motile spermatozoa. The maturity and fertilization status of oocytes were recorded after 16–18 h when all the inseminated oocytes were stripped from the cumulus cells.

The zygotes were transferred and cultured in the Continuous Single Culture (Irvine Scientific, USA) throughout the entire development stage. On day 3 after oocyte retrieval, cleavage-stage embryos were graded according to the Cummins' criteria (30). Top-quality embryos (grade I and II) were selected for vitrification, while suboptimal embryos (grade III and IV) were subjected to extended culture and observed up to the blastocyst stage. The Gardner and Schoolcraft scoring system (31) was then applied to select morphologically good blastocysts (grade ≥ 3BC) for vitrification on day 5 or 6.

### Endometrium Preparation and Frozen Embryo Transfer

Endometrium preparation and frozen embryo transfer (FET) were performed as previously described (11). Briefly, natural cycle was applied to patients with regular menstrual cycles, while patients with irregular cycles were treated with letrozole and, if necessary, in combination with hMG to stimulate mono-follicular development. Hormone replacement therapy (HRT) was recommended for patients with thin endometrium during either natural cycles or stimulated cycles. When the endometrium thickness was ≥8 mm, the transfer of day 3 or day 5/6 embryos was scheduled based on the timing of ovulation in the natural and letrozole cycle or the timing of P administration in HRT. A maximum of two embryos were transferred per patient in each FET cycle. Once a pregnancy was achieved, luteal support was continued to 10 weeks of gestation.

### Hormone Measurement

Serum concentrations of FSH, LH, E_2_, and P were routinely determined on the start day of stimulation, 5 days after stimulation, the trigger day, and the day after trigger (~10 h after the co-injection of GnRHa and hCG). Hormone levels were measured using chemiluminescence (Abbott Biologicals B.V., the Netherlands). The intra- and inter-assay coefficients of variation were 2.6 and 5.8% for FSH, 5.9 and 8.1% for LH, 6.3 and 6.4% for E_2_, and 7.9 and 10% for P, respectively. The analytical sensitivity was as follows: FSH, 0.06 IU/L; LH, 0.09 IU/L; E_2_, 10 pg/mL; and P, 0.1 ng/mL. The upper limit for E_2_ measurement was 5,000 pg/mL. If higher than the upper limit, the E_2_ levels were recorded as 5,000 pg/mL without repeating the assay after sample dilution.

### Outcome Variables and Definitions

The primary outcomes of the study were oocyte and mature oocyte yield. Other analyzed variables included IVF cycle outcomes (number of oocytes retrieved, number of MII oocytes, number of two pronuclei [2PN] oocytes, low oocyte yield, low mature oocyte yield, fertilization rate, number of top-quality embryos, and number of viable embryos), as well as pregnancy outcomes in subsequent FET cycles (positive pregnancy test rate, implantation rate, clinical pregnancy rate, ongoing pregnancy rate, pregnancy loss rate, and live birth rate).

Oocyte and mature oocyte yield were defined as the ratio of collected oocytes and MII oocytes to the number of follicles >10 mm on the day of trigger, respectively (2, 32–34). Low oocyte and mature oocyte yield were arbitrarily defined as being among the bottom 10th percentile of the distribution of oocyte yield (<52.6%) and mature oocyte yield (<40.0%), respectively. The fertilization rate was defined as the ratio of normal fertilized oocytes (2PNs) to the number of oocytes used for fertilization. Positive pregnancy test was defined as a serum β-hCG level ≥5 IU/L 14 days after FET. The implantation rate was calculated as the number of gestational sacs visualized on transvaginal ultrasound divided by the number of embryos transferred. Clinical pregnancy was identified as the presence of at least one gestational sac with or without fetal heart activity 7 weeks after FET. Ongoing pregnancy was defined as viable pregnancy at 12 weeks of gestation. Pregnancy loss was defined as pregnancies that eventuate in a spontaneous abortion or therapeutic abortion that occurred throughout pregnancy. Live birth was defined as delivery of any viable infant at 28 weeks or more of gestation.

### Statistical Analysis

Patients were categorized into five groups according to the percentage increase in E_2_ levels before and after trigger: <10.0%, 10.0–19.9%, 20.0–29.9%, 30.0–39.9%, and ≥40.0%. For continuous variables, the normality was tested by the graphical use of histograms and Q-Q plots as well as the Shapiro-Wilk test. The data were presented as mean with standard deviation (SD) and differences between groups were compared with one-way analysis of variance or Kruskal-Wallis test, as appropriate. Categorical variables were described with frequency and rate, and Chi-square test was used for comparison.

Tests for linear trend in oocyte and mature oocyte yield were performed by entering the median values of each category of E_2_ increase as a continuous variable in a general linear model. Whether or not statistical differences between groups were observed, all covariates were introduced in the final model for adjustment, including age, body mass index (BMI), infertility type (primary or secondary), infertility duration, infertility diagnosis (tubal, male, unexplained, or combined/other), additional infertility diagnosis (including PCOS and DOR), basal hormone profile (FSH, LH, E_2_, and P), AFC, duration of stimulation, total hMG dose, time interval from trigger to oocyte retrieval, duration of oocyte retrieval, fertilization method (IVF, ICSI, or IVF + ICSI) and hormone response to trigger (trigger day and post-trigger LH). The predicted mean absolute differences (MDs) and 95% confidence intervals (CIs) of oocyte and mature oocyte yield were also estimated using the lowest E_2_ increase group as reference.

For the outcomes of low oocyte and mature oocyte yield, the trend over groups was examined by Mantel-Haenszel test and a binary regression model was used to control for the aforementioned potential confounders. We also generated the receiver operating characteristic (ROC) curves to determine the predictive ability of E_2_ percentage increase after trigger. Optimal cutoff points were determined by the combination of specificity and sensitivity closest to the optimal.

All *P*-values were based on two-sided tests and the level of statistical significance was set at 0.05. Statistical analysis was performed with the Statistical Package for the Social Sciences (SPSS) (version 20.0; SPSS Inc., USA) and MedCalc (version 15.0; MedCalc Software bvba, Belgium).

## Results

A total of 7,996 patients were selected from our database who received PPOS protocol for ovarian stimulation and dual trigger for final oocyte maturation during their first IVF/ICSI cycles. Three thousand six hundred seventy-two women were categorized as normal responders, of whom 2,898 were eligible for analysis after data selection as described in the Materials and Methods section.

The number of patients with a <10.0%, 10.0–19.9%, 20.0–29.9%, 30.0–39.9%, and ≥40.0% increase in post-trigger E_2_ levels was 491 (16.9%), 671 (23.2%), 629 (21.7%), 473 (16.3%), and 634 (21.9%), respectively (Table 1). The five groups differed significantly in age, BMI, stimulation duration, total hMG dose, and duration of oocyte aspiration. The proportion of patients with DOR decreased significantly from the lowest to highest E_2_ increase group (*P*-difference = 0.002, *P*-trend = 0.001), while an opposite trend was observed in the proportion of PCOS women, total AFC and time interval from trigger to oocyte retrieval (all *P*-difference < 0.001, all *P*-trend < 0.001). There was no significant difference in infertility type, duration and diagnosis, fertilization method, and basal hormone profile except for P level.

**Table 1:** Patient characteristics according to the magnitude of E_2_ increase after dual trigger.

	**<10.0%** **(*n* = 491)**	**10.0–19.9%** **(*n* = 671)**	**20.0–29.9%** **(*n* = 629)**	**30.0–39.9%** **(*n* = 473)**	**≥40.0%** **(*n* = 634)**	***P-*value for difference**
Age (years)	32.36 ± 4.58	32.43 ± 4.53	31.93 ± 4.52	31.88 ± 4.35	31.54 ± 4.55	0.003
Body mass index (kg/m^2^)	21.58 ± 2.80	21.84 ± 3.07	21.90 ± 3.02	22.15 ± 3.33	22.51 ± 3.10	<0.001
Primary infertility, *n* (%)	250 (50.9)	346 (51.6)	326 (51.8)	263 (55.6)	338 (53.3)	0.586
Duration of infertility (years)	3.5 ± 2.8	3.4 ± 3.0	3.4 ± 2.6	3.4 ± 2.7	3.4 ± 2.6	0.847
**Infertility diagnosis**, ***n*** **(%)**						
Tubal factor	230 (46.8)	319 (47.5)	308 (49.0)	225 (47.6)	294 (46.4)	0.917
Male factor	72 (14.7)	86 (12.8)	78 (12.4)	62 (13.1)	88 (13.9)	
Unexplained	66 (13.4)	102 (15.2)	84 (13.4)	60 (12.7)	77 (12.1)	
Combined/other	123 (25.1)	164 (24.4)	159 (25.3)	126 (26.6)	175 (27.6)	
Infertility diagnosis including PCOS, *n* (%)	13 (2.6)	23 (3.4)	31 (4.9)	32 (6.8)	58 (9.1)	<0.001
Infertility diagnosis including DOR, *n* (%)	43 (8.8)	44 (6.6)	37 (5.9)	23 (4.9)	20 (3.2)	0.002
**Basal hormone profile**						
FSH (mIU/mL)	6.10 ± 1.45	6.09 ± 1.44	6.03 ± 1.44	6.03 ± 1.43	5.89 ± 1.35	0.080
LH (mIU/mL)	3.59 ± 1.60	3.44 ± 1.47	3.48 ± 1.52	3.41 ± 1.66	3.59 ± 2.05	0.266
E_2_ (pg/mL)	35.3 ± 14.1	34.4 ± 13.8	34.3 ± 14.3	33.5 ± 14.0	33.6 ± 14.7	0.068
P (ng/mL)	0.28 ± 0.11	0.29 ± 0.11	0.27 ± 0.11	0.27 ± 0.11	0.28 ± 0.11	0.024
Total antral follicle count	9.6 ± 4.2	10.4 ± 4.7	10.5 ± 4.7	11.1 ± 4.9	12.1 ± 6.0	<0.001
Stimulation duration (days)	8.9 ± 1.3	8.9 ± 1.4	8.7 ± 1.2	8.8 ± 1.3	8.7 ± 1.4	0.007
Total hMG dose (IU)	1917.9 ± 328.8	1961.6 ± 353.1	1923.4 ± 316.2	1932.1 ± 355.9	1902.7 ± 359.9	<0.001
Time interval from trigger to oocyte aspiration (hours)	36.48 ± 0.73	36.66 ± 0.75	36.74 ± 0.77	36.83 ± 0.77	36.91 ± 0.85	<0.001
Duration of oocyte aspiration (minutes)	9.19 ± 1.89	9.55 ± 1.90	9.61 ± 2.13	9.31 ± 2.05	9.60 ± 1.84	0.018
**Fertilization method**, ***n*** **(%)**						
IVF	297 (60.5)	424 (63.2)	377 (59.9)	291 (61.5)	380 (59.9)	0.324
ICSI	127 (25.9)	165 (24.6)	177 (28.1)	125 (26.4)	152 (24.0)	
IVF+ICSI	67 (13.6)	82 (12.2)	75 (11.9)	57 (12.1)	102 (16.1)	

*Values are presented as mean ± SD or number (percentage). E_2_, estradiol; PCOS, polycystic ovarian syndrome; DOR, diminished ovarian reserve; FSH, follicular stimulating hormone; LH, luteinizing hormone; P, progesterone; hMG, human menopausal gonadotropin; IVF, in vitro fertilization; ICSI, intracytoplasmic sperm injection*.

The E_2_ levels on the day of trigger differed among the five groups, with significantly lower levels in the higher E_2_ increase group (*P*-difference < 0.001, *P*-trend < 0.001) (Table 2). On the contrary, post-trigger E_2_ levels increased significantly over groups (*P*-difference < 0.001, *P*-trend < 0.001). No significant differences were detected when trigger day LH and post-trigger LH levels were analyzed (*P*-difference = 0.494 and 0.572, respectively).

**Table 2:** IVF cycle outcomes according to the magnitude of E_2_ increase after dual trigger.

	**<10.0%** **(*n* = 491)**	**10.0–19.9%** **(*n* = 671)**	**20.0–29.9%** **(*n* = 629)**	**30.0–39.9%** **(*n* = 473)**	**≥40.0%** **(*n* = 634)**	***P-*value for difference**
**Hormone response to trigger**						
Trigger day E_2_ (pg/mL)	2612.7 ± 824.8	2575.1 ± 824.7	2475.0 ± 806.3	2392.5 ± 770.2	2011.9 ± 707.5	<0.001
Post-trigger E_2_ (pg/mL)	2755.1 ± 877.1	2964.5 ± 953.0	3087.7 ± 1011.0	3222.5 ± 1038.3	3136.4 ± 1057.4	<0.001
Trigger day LH (mIU/mL)	1.88 ± 1.51	1.87 ± 1.23	1.88 ± 1.19	1.87 ± 1.39	1.87 ± 1.47	0.494
Post-trigger LH (mIU/mL)	51.41 ± 19.44	50.43 ± 19.69	51.97 ± 20.18	51.30 ± 21.51	52.31 ± 21.72	0.572
E_2_ absolute difference (pg/mL)	142.4 ± 90.6	389.4 ± 146.6	612.7 ± 215.4	830.0 ± 275.8	1124.5 ± 432.9	<0.001
E_2_ percent difference (%)	5.4 ± 2.8	15.1 ± 2.8	24.7 ± 2.8	34.7 ± 2.9	57.6 ± 18.0	<0.001
**IVF cycle outcomes**						
No. of follicles on trigger day	11.6 ± 4.0	12.1 ± 4.3	12.2 ± 4.5	12.6 ± 4.2	13.1 ± 4.9	<0.001
No. of follicles >10 mm on trigger day	10.0 ± 3.8	10.2 ± 3.8	10.4 ± 4.0	10.7 ± 3.8	10.9 ± 4.2	0.006
No. of oocytes retrieved	8.4 ± 3.5	8.6 ± 3.5	8.8 ± 3.8	8.6 ± 3.3	8.3 ± 3.3	0.329
No. of MII oocytes	7.3 ± 3.2	7.1 ± 3.3	7.4 ± 3.5	7.3 ± 3.0	7.0 ± 3.0	0.232
No. of 2PN oocytes	6.0 ± 3.0	5.9 ± 3.0	6.0 ± 3.2	6.0 ± 2.8	5.7 ± 2.8	0.290
Oocyte yield (%)	88.2 ± 28.5	87.7 ± 30.5	87.8 ± 29.6	84.5 ± 26.8	81.8 ± 30.0	<0.001
Mature oocyte yield (%)	76.1 ± 28.0	73.6 ± 30.1	73.5 ± 28.2	72.0 ± 25.5	67.9 ± 26.5	<0.001
Low oocyte yield, *n* (%)	35 (7.1)	55 (8.2)	61 (9.7)	53 (11.2)	86 (13.6)	0.002
Low mature oocyte yield, *n* (%)	30 (6.1)	54 (8.0)	56 (8.9)	28 (5.9)	77 (12.1)	<0.001
Fertilization rate (%)	78.7 ± 21.6	77.6 ± 23.7	78.2 ± 23.1	77.2 ± 20.7	77.0 ± 23.0	0.349
No. of top-quality embryos	3.0 ± 2.3	2.8 ± 2.2	2.8 ± 2.3	2.8 ± 2.1	2.7 ± 2.1	0.263
No. of viable embryos	3.4 ± 2.2	3.2 ± 2.2	3.3 ± 2.3	3.3 ± 2.1	3.1 ± 2.1	0.343

*Values are presented as mean ± SD or number (percentage). IVF, in vitro fertilization; E_2_, estradiol; LH, luteinizing hormone; MII, metaphase II; 2PN, two pronuclei*.

**Figure 1:**
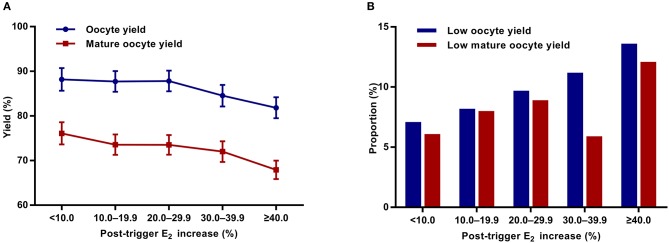
Association between post-trigger E_2_ increase and **(A)** oocyte and mature oocyte yield, as well as **(B)** proportion of low oocyte and mature yield. E_2_, estradiol.

**Figure 2:**
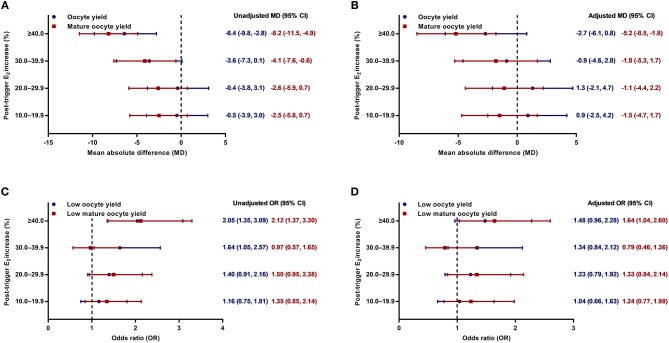
**(A,B)** Unadjusted and adjusted mean absolute differences (MDs) for oocyte and mature oocyte yield by the magnitude of post-trigger E_2_ increase compared with the <10.0% rise group. **(C,D)** Unadjusted and adjusted odds ratios (ORs) for low oocyte and mature oocyte yield by the magnitude of post-trigger E_2_ increase compared with the <10.0% rise group. E_2_, estradiol.

Although there was a positive association between the post-trigger E_2_ increase and the number of follicles (*P*-difference < 0.001, *P*-trend < 0.001) and follicles >10 mm (*P*-difference = 0.006, *P*-trend < 0.001) on trigger day, we did not find statistically significant results in the number of oocytes and MII oocyte retrieved (*P*-difference = 0.329 and 0.232, respectively) (Table 2). As expected, there was a general decline in both oocyte and mature oocyte yield with a higher E_2_ increase (all *P*-difference < 0.001, all *P*-trend < 0.001), and the trends remained after adjusting for cofounders (adjusted *P*-trend = 0.033 and 0.002, respectively) (Figure 1A). Relative to those with a <10.0% increase in post-trigger E_2_ levels, women with a ≥40.0% increase had significantly lower oocyte and mature oocyte yield [unadjusted MD [95% CI] = −6.4 [−9.8, −2.8] and −8.2 [−11.5, −4.9], respectively] (Figure 2A). The difference failed to reach statistical significance in oocyte yield [adjusted MD [95% CI] = −2.7 [−6.1, 0.8], *P* = 0.133], but was maintained regarding mature oocyte yield [adjusted MD [95% CI] = −5.2 [−8.2, −1.8], *P* = 0.002] after adjustment (Figure 2B).

A significant positive trend was observed with the magnitude of E_2_ increment after trigger when looking at the proportion of low oocyte yield (*P*-difference = 0.002, *P*-trend < 0.001) and low mature oocyte yield (*P*-difference < 0.001, *P*-trend = 0.004) (Figure 1B). Compared with patients with a <10.0% E_2_ rise, a ≥40.0% rise conferred a 2-fold increase in the odds of both low oocyte and mature oocyte yield [unadjusted OR [95% CI] = 2.05 [1.35, 3.09] and 2.12 [1.37, 3.30], respectively] (Figure 2C). Multivariable logistic regression demonstrated that the higher risk of low mature oocyte yield remained in the ≥40.0% increase group [adjusted OR [95% CI] = 1.64 [1.04, 2.60], *P* = 0.035], but was not statistically significant for low oocyte yield [adjusted OR [95% CI] = 1.48 [0.96, 2.28], *P* = 0.077] (Figure 2D).

The ROC analysis revealed that the post-trigger E_2_ percentage increase had a modest predictive accuracy at best for low oocyte and mature oocyte yield, with an area under the ROC curve (AUC) of 0.579 [95% CI = [0.561, 0.597], *P* < 0.001] and 0.560 [95% CI = [0.542, 0.578], *P* = 0.002], respectively (Supplementary Figure 1). The optimal cutoff value of E_2_ percentage change for low oocyte yield prediction was 22.9% with a sensitivity of 65.5% and a specificity of 48.0%, while the threshold of 40.7% was shown to predict low mature oocyte yield with a sensitivity of 31.4% and a specificity of 80.1%.

There was no significant difference between the five groups regarding the number of 2PN oocytes, fertilization rate, number of top-quality embryos, and number of viable embryos (Table 2). The pregnancy outcomes of 1,478 patients were further analyzed who completed a total of 1,942 FET cycles with embryos originating from different groups of post-trigger E_2_ increase. Likewise, we did not find statistically significant difference in the implantation rate across groups (*P*-difference = 0.066) (Supplementary Table 1). The rates of positive pregnancy test, clinical pregnancy, ongoing pregnancy, pregnancy loss, and live birth were also demonstrably similar (unadjusted *P*-difference = 0.082, 0.104, 0.187, 0.089, and 0.110, respectively), which remined consistent after adjusting for patient age, BMI, infertility type, duration, and diagnosis, additional infertility diagnosis (including PCOS and DOR), number of embryos transferred, embryo stage at transfer, endometrial preparation, and endometrial thickness (adjusted *P*-difference = 0.264, 0.458, 0.753, 0.128, and 0.551, respectively).

## Discussion

In this large retrospective study, we showed for the first time that both oocyte and mature oocyte yield decreased gradually with a higher E_2_ rise after dual trigger in normal responders. A post-trigger E_2_ increase of 40% could be identified as an appropriate threshold, above which mature oocyte yield is significantly reduced, and the risk of low mature oocyte yield tend to be higher. Furthermore, our study demonstrated comparable pregnancy outcomes in the subsequent FET cycles with embryos originating from groups of different magnitude of E_2_ increment.

When quantifying the efficacy of triggering, the number of oocytes and mature oocytes retrieved are frequently utilized for evaluation. This approach can be validating if an equal number of follicles are available for aspiration among each group, especially in powered prospective randomized studies. However, it relies heavily on individual patient factors and hence may not be appropriate in actual clinical settings (2). Other frequently reported measures include the oocyte retrieval rate and mature oocyte rate, which refer to the ratio of oocytes retrieved to the number of follicles aspirated and the proportion of MII oocytes to the number of collected oocytes, respectively. Since immature oocytes derived from smaller follicles often pose difficulties on retrieval, insufficient triggering could cause a reduction in both the denominator and numerator, thus making the measures less reliable in assessment of trigger efficacy (2). Therefore, in the present study, we used the oocyte and mature oocyte yield as primary outcomes where the number of retrieved oocytes and mature oocytes were corrected for the number of follicles >10 mm on the day of trigger (2, 32–34). The size of follicles was determined in accordance with previous studies from others and ours (2, 11, 32–35), while a denominator of follicles >14 mm was also applied elsewhere (33, 36, 37). Although follicles with a diameter of 16–22 mm on the day of oocyte retrieval are most likely to yield oocytes (20, 38, 39), there are limited data to justify specific categories of follicle size on trigger day for estimation (19). Only one group (19) very recently suggested that follicles 12–19 mm on the day of trigger contributed the most to the number of oocytes and mature oocytes retrieved, but interventional studies are still lacking in proving its prognostic value.

The significance of changes in post-trigger E_2_ levels has been evaluated previously with a major focus on pregnancy outcome following IVF-ET cycles, although no general agreement has been reached on it (13–18). In 1986, Laufer et al. (13) first reported that patients with an increase in E_2_ levels after hCG administration had significantly more oocytes retrieved laparoscopically but demonstrated no difference in fertilization rates. This was challenged later by a prospective cohort study (14) showing that an E_2_ increase of >10% had no effect on the number of mature oocytes but associated with a significantly lower fertilization rate. In 2007, Chiasson et al. (15) conducted a retrospective analysis of 844 IVF cycles and found that patients with >30% increase in E_2_ level after trigger had more oocytes and mature oocytes retrieved than those with 10–30% increase or ±10% change. Two subsequent large retrospective studies (16, 18), either in stimulated or natural IVF cycle treatment, drew the same result that a higher number of oocytes could be expected without interfering the fertilization rates when an E_2_ rise of >10% was observed. However, robustness of these results was not tested after properly controlling for potential confounding factors (i.e., AFC). All the studies did not analyze the outcomes of oocyte and mature oocyte yield, nor did the relationship between the degree of E_2_ increase after trigger and outcome measures. Unignorable problems also exist in some of the studies, including relatively small sample size (13, 14), heterogeneous patient population (15), and ununiform stimulation/trigger protocols (16–18). Moreover, fresh embryo transfer ensued following IVF cycles in most studies, so it remains unknown whether the difference in pregnancy outcomes derives from suboptimal oocyte/embryo quality or impaired endometrial receptivity caused by supraphysiological hormone concentrations during COS (40).

In an effort to address these shortcomings, our study was designed with restrictions in the homogeneous population (normal responders), the standard COS treatment (PPOS protocol), and the same trigger method (dual trigger). Based on the large sample size of nearly 3,000 first IVF/ICSI cycles within a single center, we observed no difference in the number of oocytes retrieved and mature oocytes as well as the fertilization rates. Instead, the findings showed a trend toward significantly lower oocyte and mature oocyte yield with the gradual increment in post-trigger E_2_ level. This decline was more evident when the increase exceeded 40% and remained stable even after adjusting for a number of important confounders. Since the freeze-all policy was applied for all the patients, the results of similar pregnancy outcomes imply that E_2_ increase after trigger may not have detrimental effects on the quality of oocytes or the developmental potential of embryos.

The underlying mechanism for the association between a higher post-trigger E_2_ rise and a reduction in oocyte and mature oocyte yield is unclear. The two-cell/two-gonadotropin hypothesis reveals that LH acts on the theca-interstitial cells to promote the biosynthesis of androgens, which are further aromatized into estrogens by FSH-inducible granulosa cells (41). During the follicular phase of natural menstrual cycles, E_2_ production rises continuously with the growth, and development of follicles (41). This is presumed to be associated with an increase in the LH/CG receptor expression (42) as well as the induction of the key steroidogenic enzyme 17α-hydroxylase/17,20-desmolase (43). However, after the initiation of midcycle LH surge, the receptor number and mRNA levels decrease abruptly due to an LH-induced increase in intracellular cyclic adenosine monophosphate (cAMP) concentration (44). LH also stimulates the expression of P receptors on the granulosa cells of the dominant follicle (45), promoting luteinization and early P elevation, which slows granulosa cell proliferation (46). Both effects consequently result in a decline of serum E_2_ level in combination. In contrast, multiple follicles develop during IVF cycles and final oocyte maturation triggering is initiated as soon as the leading follicles meet the requisite size. The residual follicles of smaller sizes, however, may not be sensitive to the LH-like exposure yet, which causes a lower oocyte and mature oocyte yield and conversely facilitates the E_2_ generation by contributing to the expression of LH receptor in theca cells (47). A higher increase in circulating E_2_ level after trigger can therefore serve as a marker of a more asynchronized cohort of follicular development in non-optimal situations. Further investigations are warranted to examine the explanation with stronger direct evidence.

As in the course of natural ovulatory cycle, the LH activity of hCG and/or GnRHa induce the cumulus expansion, COC disassociation, and germinal vesicle breakdown (2, 3). Therefore, scheduling the time interval from oocyte maturation triggering to oocyte retrieval precisely is important in guaranteeing the optimal action duration of trigger agents on the follicles developed. Follicles can grow “postmature” or ovulate prematurely if the interval is too long, while a short interval is not sufficient for oocyte maturation and makes retrieval more difficult. However, previous studies (34, 48, 49) have failed to identify an optimal interval and produced controversial results on whether prolongation of the interval would improve oocyte retrieval outcomes. One possible explanation may be that none of these studies took the follicle size profiles on trigger day or the hormonal response after trigger into account. Indeed, a serum LH level of ≤ 15.0 IU/L post-trigger with GnRHa has been shown to be associated with a dramatically lower oocyte retrieval rate, smaller number of mature oocytes, and higher incidence of empty follicle syndrome (22, 23). Moreover, a recent study from our group (18) suggests that emergency follicle aspiration should be performed in natural/unstimulated cycles with a >23% decline in preovulatory E_2_ level, which could help reduce the occurrence of premature ovulation and avoid IVF cycle cancellation. However, it remains to be further elucidated whether extension of the time interval would exert beneficial effects on the oocyte and mature oocyte yield when an increased E_2_ level is measured after trigger.

A major weakness of the current study relies on its retrospective and non-randomized design, although the ascertainment and recall bias were minimized because all the data were gathered and documented in the computerized database. In this regard, we only enrolled patients undergoing the first IVF cycles and meticulously screened patients with strict criteria for inclusion. However, other possible unknown or unavailable confounding factors were not included for adjustment such as basal androgen level, and the application of our findings should not be extrapolated to patients with high or low ovarian response. Another limitation is that the upper limit of E_2_ measurement is 5,000 pg/mL in our laboratory. If higher, the E_2_ level was recorded as 5,000 pg/mL without dilution of serum samples for repeating the assay. Therefore, patients with the upper limit of E_2_ concentration on the day after trigger were excluded to optimize the data quality and accuracy. Despite the relatively small proportion (4.9%), we recognize this limitation since the potential selection bias might be present. Lastly, analysis of pregnancy outcomes in the present study was based on the criteria of *per transfer* without calculating cumulative live birth rate after a complete IVF cycle. Given that the cumulative live birth rate increased with ovarian response represented by the number of oocytes retrieved (50, 51), we hold that further studies would be necessary to explore the association between post-trigger E_2_ increase and pregnancy outcomes in the context of *per patient*.

## Conclusion

In conclusion, our study suggests that a higher post-trigger E_2_ increase is associated with a lower oocyte and mature oocyte yield among normal responders. Therefore, the magnitude of E_2_ rise after trigger can be used as another parameter by clinicians for patient counseling and prediction of oocyte retrieval outcomes. Further studies are needed to explore the underlying mechanism behind the relationship, and to investigate the efficacy of individualized time interval from trigger to oocyte retrieval based on the magnitude of post-trigger E_2_ rise.

## Data Availability Statement

The datasets generated for this study are available on request to the corresponding author.

## Ethics Statement

The study protocol was approved by the Ethics Committee (Institutional Review Board) of Shanghai Ninth People's Hospital affiliated with Shanghai Jiao Tong University School of Medicine (No: 2017-17). All subjects gave written informed consent in accordance with the Declaration of Helsinki.

## Author Contributions

JH, RC, and YK contributed to the conception and design of the study. JH, XL, JL, NW, HG, and QL were responsible for data acquisition and checking. JH and XL performed the data analysis, interpretation, and manuscript drafting. RC and YK supervised the project administration. All authors read, revised and approved the final manuscript.

### Conflict of Interest

The authors declare that the research was conducted in the absence of any commercial or financial relationships that could be construed as a potential conflict of interest.

## Supplementary Material

Supplementary Figure 1 Receiver operating characteristic curves of post-trigger E_2_ percentage increase for the prediction of **(A)** low oocyte yield and **(B)** low mature oocyte yield. The diagonal line is the reference line of no discrimination (area under the curve = 0.5). The marked red and green points correspond with the optimal cutoff value and the criterion value of 40%, respectively. E_2_, estradiol.

Supplementary Table 1 Pregnancy outcomes of frozen-thawed embryos originating from groups stratified by the magnitude of E2 increase after dual trigger.

## References

[1] Voronina E Wessel GM. The regulation of oocyte maturation. Curr Top Dev Biol. (2003) 58:53–110. 10.1016/S0070-2153(03)58003-6 14711013

[2] Abbara A Clarke SA Dhillo WS. Novel concepts for inducing final oocyte maturation in *in vitro* fertilization treatment. Endocr Rev. (2018) 39:593–628. 10.1210/er.2017-00236 29982525 PMC6173475

[3] Castillo JC Humaidan P Bernabeu R. Pharmaceutical options for triggering of final oocyte maturation in ART. Biomed Res Int. (2014) 2014:580171. 10.1155/2014/580171 25133168 PMC4123594

[4] Damewood MD Shen W Zacur HA Schlaff WD Rock JA Wallach EE. Disappearance of exogenously administered human chorionic gonadotropin. Fertil Steril. (1989) 52:398–400. 10.1016/S0015-0282(16)60906-8 2776893

[5] Itskovitz J Boldes R Levron J Erlik Y Kahana L Brandes JM. Induction of preovulatory luteinizing hormone surge and prevention of ovarian hyperstimulation syndrome by gonadotropin-releasing hormone agonist. Fertil Steril. (1991) 56:213–20. 10.1016/S0015-0282(16)54474-4 1906406

[6] Humaidan P Bredkjaer HE Bungum L Bungum M Grondahl ML Westergaard L GnRH agonist (buserelin) or hCG for ovulation induction in GnRH antagonist IVF/ICSI cycles: a prospective randomized study. Hum Reprod. (2005) 20:1213–20. 10.1093/humrep/deh765 15760966

[7] Shapiro BS Daneshmand ST Garner FC Aguirre M Hudson C. Comparison of “triggers” using leuprolide acetate alone or in combination with low-dose human chorionic gonadotropin. Fertil Steril. (2011) 95:2715–7. 10.1016/j.fertnstert.2011.03.109 21550042

[8] Griffin D Feinn R Engmann L Nulsen J Budinetz T Benadiva C. Dual trigger with gonadotropin-releasing hormone agonist and standard dose human chorionic gonadotropin to improve oocyte maturity rates. Fertil Steril. (2014) 102:405–9. 10.1016/j.fertnstert.2014.04.028 24842671

[9] Hillier SG Reichert LE Jr Van Hall EV. Control of preovulatory follicular estrogen biosynthesis in the human ovary. J Clin Endocrinol Metab. (1981) 52:847–56. 10.1210/jcem-52-5-847 6785289

[10] Kuang Y Hong Q Chen Q Lyu Q Ai A Fu Y. Luteal-phase ovarian stimulation is feasible for producing competent oocytes in women undergoing *in vitro* fertilization/intracytoplasmic sperm injection treatment, with optimal pregnancy outcomes in frozen-thawed embryo transfer cycles. Fertil Steril. (2014) 101:105–11. 10.1016/j.fertnstert.2013.09.007 24161646

[11] Kuang Y Chen Q Fu Y Wang Y Hong Q Lyu Q. Medroxyprogesterone acetate is an effective oral alternative for preventing premature luteinizing hormone surges in women undergoing controlled ovarian hyperstimulation for *in vitro* fertilization. Fertil Steril. (2015) 104:62–70.e3. 10.1016/j.fertnstert.2015.03.022 25956370

[12] Massin N. New stimulation regimens: endogenous and exogenous progesterone use to block the LH surge during ovarian stimulation for IVF. Hum Reprod Update. (2017) 23:211–20. 10.1093/humupd/dmw047 28062551

[13] Laufer N DeCherney AH Tarlatzis BC Naftolin F. The association between preovulatory serum 17 beta-estradiol pattern and conception in human menopausal gonadotropin-human chorionic gonadotropin stimulation. Fertil Steril. (1986) 46:73–6. 10.1016/S0015-0282(16)49460-4 3087791

[14] Meyer WR Beyler SA Baker ST Somkuti SG Lowden DA Grainger DA. Value of estradiol response after human chorionic gonadotropin administration in predicting *in vitro* fertilization success. Fertil Steril. (1999) 72:542–5. 10.1016/S0015-0282(99)00281-2 10519632

[15] Chiasson MD Bates GW Robinson RD Arthur NJ Propst AM Measuring estradiol levels after human chorionic gonadotropin administration for *in vitro* fertilization is not clinically useful. Fertil Steril. (2007) 87:448–50. 10.1016/j.fertnstert.2006.06.024 17084395

[16] Kondapalli LA Molinaro TA Sammel MD Dokras A. A decrease in serum estradiol levels after human chorionic gonadotrophin administration predicts significantly lower clinical pregnancy and live birth rates in *in vitro* fertilization cycles. Hum Reprod. (2012) 27:2690–7. 10.1093/humrep/des216 22752608 PMC3415290

[17] Huang R Fang C Wang N Li L Yi Y Liang X. Serum estradiol level change after human chorionic gonadotropin administration had no correlation with live birth rate in IVF cycles. Eur J Obstet Gynecol Reprod Biol. (2014) 178:177–82. 10.1016/j.ejogrb.2014.02.040 24862918

[18] Lu X Khor S Zhu Q Sun L Wang Y Chen Q. Decrease in preovulatory serum estradiol is a valuable marker for predicting premature ovulation in natural/unstimulated *in vitro* fertilization cycle. J Ovarian Res. (2018) 11:96. 10.1186/s13048-018-0469-x 30463583 PMC6247609

[19] Abbara A Vuong LN Ho VNA Clarke SA Jeffers L Comninos AN. Follicle size on day of trigger most likely to yield a mature oocyte. Front Endocrinol. (2018) 9:193. 10.3389/fendo.2018.00193 29743877 PMC5930292

[20] Revelli A Martiny G Delle Piane L Benedetto C Rinaudo P Tur-Kaspa I. A critical review of bi-dimensional and three-dimensional ultrasound techniques to monitor follicle growth: do they help improving IVF outcome? Reprod Biol Endocrinol. (2014) 12:107. 10.1186/1477-7827-12-107 25420733 PMC4255967

[21] Lin MH Wu FS Lee RK Li SH Lin SY Hwu YM. Dual trigger with combination of gonadotropin-releasing hormone agonist and human chorionic gonadotropin significantly improves the live-birth rate for normal responders in GnRH-antagonist cycles. Fertil Steril. (2013) 100:1296–302. 10.1016/j.fertnstert.2013.07.1976 23993928

[22] Chen SL Ye DS Chen X Yang XH Zheng HY Tang Y. Circulating luteinizing hormone level after triggering oocyte maturation with GnRH agonist may predict oocyte yield in flexible GnRH antagonist protocol. Hum Reprod. (2012) 27:1351–6. 10.1093/humrep/des049 22419746

[23] Lu X Hong Q Sun L Chen Q Fu Y Ai A. Dual trigger for final oocyte maturation improves the oocyte retrieval rate of suboptimal responders to gonadotropin-releasing hormone agonist. Fertil Steril. (2016) 106:1356–62. 10.1016/j.fertnstert.2016.07.1068 27490046

[24] Feng R Sang Q Kuang Y Sun X Yan Z Zhang S. Mutations in TUBB8 and human oocyte meiotic arrest. N Engl J Med. (2016) 374:223–32. 10.1056/NEJMoa1510791 26789871 PMC4767273

[25] Sang Q Li B Kuang Y Wang X Zhang Z Chen B. Homozygous mutations in WEE2 cause fertilization failure and female infertility. Am J Hum Genet. (2018) 102:649–57. 10.1016/j.ajhg.2018.02.015 29606300 PMC5985286

[26] Chen B Zhang Z Sun X Kuang Y Mao X Wang X. Biallelic mutations in PATL2 cause female infertility characterized by oocyte maturation arrest. Am J Hum Genet. (2017) 101:609–15. 10.1016/j.ajhg.2017.08.018 28965849 PMC5630194

[27] Rotterdam ESHRE/ASRM-Sponsored PCOS Consensus Workshop Group Revised 2003 consensus on diagnostic criteria and long-term health risks related to polycystic ovary syndrome. Fertil Steril. (2004) 81:19–25. 10.1016/j.fertnstert.2003.10.004 14711538

[28] Molinaro TA Shaunik A Lin K Sammel MD Barnhart KT. A strict infertility diagnosis has poor agreement with the clinical diagnosis entered into the Society for Assisted Reproductive Technology registry. Fertil Steril. (2009) 92:2088–90. 10.1016/j.fertnstert.2009.05.082 19635611 PMC2789855

[29] Zhang J Mao X Wang Y Chen Q Lu X Hong Q. Neonatal outcomes and congenital malformations in children born after human menopausal gonadotropin and medroxyprogesterone acetate treatment cycles. Arch Gynecol Obstet. (2017) 296:1207–17. 10.1007/s00404-017-4537-z 28948397

[30] Cummins JM Breen TM Harrison KL Shaw JM Wilson LM Hennessey JF. A formula for scoring human embryo growth rates in *in vitro* fertilization: its value in predicting pregnancy and in comparison with visual estimates of embryo quality. J In vitro Fert Embryo Transf . (1986) 3:284–95. 10.1007/BF01133388 3783014

[31] Gardner DK Schoolcraft WB *In vitro* culture of human blastocyst. In: Jansen R Mortimer D, editors. Towards Reproductive Certainty: Infertility and Genetics Beyond 1999. Carnforth: Parthenon Press (1999). p. 378–88.

[32] Shapiro BS Daneshmand ST Restrepo H Garner FC Aguirre M Hudson C. Efficacy of induced luteinizing hormone surge after “trigger” with gonadotropin-releasing hormone agonist. Fertil Steril. (2011) 95:826–8. 10.1016/j.fertnstert.2010.09.009 20961539

[33] Haas J Zilberberg E Dar S Kedem A Machtinger R Orvieto R. Co-administration of GnRH-agonist and hCG for final oocyte maturation (double trigger) in patients with low number of oocytes retrieved per number of preovulatory follicles–a preliminary report. J Ovarian Res. (2014) 7:77. 10.1186/1757-2215-7-77 25296696 PMC4237863

[34] Bosdou JK Kolibianakis EM Venetis CA Zepiridis L Chatzimeletiou K Makedos A. Is the time interval between HCG administration and oocyte retrieval associated with oocyte retrieval rate? Reprod Biomed Online. (2015) 31:625–32. 10.1016/j.rbmo.2015.08.005 26387934

[35] Hu X Luo Y Huang K Li Y Xu Y Zhou C. New perspectives on criteria for the determination of HCG trigger timing in GnRH antagonist cycles. Medicine. (2016) 95:e3691. 10.1097/MD.0000000000003691 27196479 PMC4902421

[36] Abbara A Jayasena CN Christopoulos G Narayanaswamy S Izzi-Engbeaya C Nijher GM. Efficacy of kisspeptin-54 to trigger oocyte maturation in women at high risk of Ovarian Hyperstimulation Syndrome (OHSS) during *in vitro* Fertilization (IVF) therapy. J Clin Endocrinol Metab. (2015) 100:3322–31. 10.1210/jc.2015-2332 26192876 PMC4570165

[37] Jayasena CN Abbara A Comninos AN Nijher GM Christopoulos G Narayanaswamy S. Kisspeptin-54 triggers egg maturation in women undergoing *in vitro* fertilization. J Clin Invest. (2014) 124:3667–77. 10.1172/JCI75730 25036713 PMC4109525

[38] Ectors FJ Vanderzwalmen P Van Hoeck J Nijs M Verhaegen G Delvigne A Relationship of human follicular diameter with oocyte fertilization and development after *in-vitro* fertilization or intracytoplasmic sperm injection. Hum Reprod. (1997) 12:2002–5. 10.1093/humrep/12.9.2002 9363720

[39] Dubey AK Wang HA Duffy P Penzias AS. The correlation between follicular measurements, oocyte morphology, and fertilization rates in an *in vitro* fertilization program. Fertil Steril. (1995) 64:787–90. 10.1016/S0015-0282(16)57855-8 7672151

[40] Bourgain C Devroey P. The endometrium in stimulated cycles for IVF. Hum Reprod Update. (2003) 9:515–22. 10.1093/humupd/dmg045 14714588

[41] Shoham Z Schachter M. Estrogen biosynthesis–regulation, action, remote effects, and value of monitoring in ovarian stimulation cycles. Fertil Steril. (1996) 65:687–701. 10.1016/S0015-0282(16)58197-7 8654622

[42] Ireland JJ Richards JS. A previously underscribed role for luteinizing hormone (LH:hCG) on follicular cell differentiation. Endocrinology. (1978) 102:1458–65. 10.1210/endo-102-5-1458 217624

[43] Bogovich K Richards JS. Androgen biosynthesis in developing ovarian follicles: evidence that luteinizing hormone regulates thecal 17 alpha-hydroxylase and C17-20-lyase activities. Endocrinology. (1982) 111:1201–8. 10.1210/endo-111-4-1201 6288350

[44] Segaloff DL Wang HY Richards JS. Hormonal regulation of luteinizing hormone/chorionic gonadotropin receptor mRNA in rat ovarian cells during follicular development and luteinization. Mol Endocrinol. (1990) 4:1856–65. 10.1210/mend-4-12-1856 2127956

[45] Hild-Petito S Stouffer RL Brenner RM. Immunocytochemical localization of estradiol and progesterone receptors in the monkey ovary throughout the menstrual cycle. Endocrinology. (1988) 123:2896–905. 10.1210/endo-123-6-2896 3197647

[46] Chaffkin LM Luciano AA Peluso JJ. Progesterone as an autocrine/paracrine regulator of human granulosa cell proliferation. J Clin Endocrinol Metab. (1992) 75:1404–8. 10.1210/jc.75.6.1404 1464640

[47] Jia XC Hsueh AJ. Homologous regulation of hormone receptors: luteinizing hormone increases its own receptors in cultured rat granulosa cells. Endocrinology. (1984) 115:2433–9. 10.1210/endo-115-6-2433 6094158

[48] Weiss A Neril R Geslevich J Lavee M Beck-Fruchter R Golan J. Lag time from ovulation trigger to oocyte aspiration and oocyte maturity in assisted reproductive technology cycles: a retrospective study. Fertil Steril. (2014) 102:419–23. 10.1016/j.fertnstert.2014.04.041 24880653

[49] Wang W Zhang XH Wang WH Liu YL Zhao LH Xue SL. The time interval between hCG priming and oocyte retrieval in ART program: a meta-analysis. J Assist Reprod Genet. (2011) 28:901–10. 10.1007/s10815-011-9613-x 21792666 PMC3220445

[50] Ji J Liu Y Tong XH Luo L Ma J Chen Z. The optimum number of oocytes in IVF treatment: an analysis of 2455 cycles in China. Hum Reprod. (2013) 28:2728–34. 10.1093/humrep/det303 23887075

[51] Zhu Q Chen Q Wang L Lu X Lyu Q Wang Y. Live birth rates in the first complete IVF cycle among 20 687 women using a freeze-all strategy. Hum Reprod. (2018) 33:924–9. 10.1093/humrep/dey044 29534178

