# Supplementary figures and images for: A Higher Estradiol Rise After Dual Trigger in Progestin-Primed Ovarian Stimulation Is Associated With a Lower Oocyte and Mature Oocyte Yield in Normal Responders
Source: Front Endocrinol (Lausanne). 2019 Oct 9;10:696. doi: 10.3389/fendo.2019.00696 (PMC6794366; doi:10.3389/fendo.2019.00696)

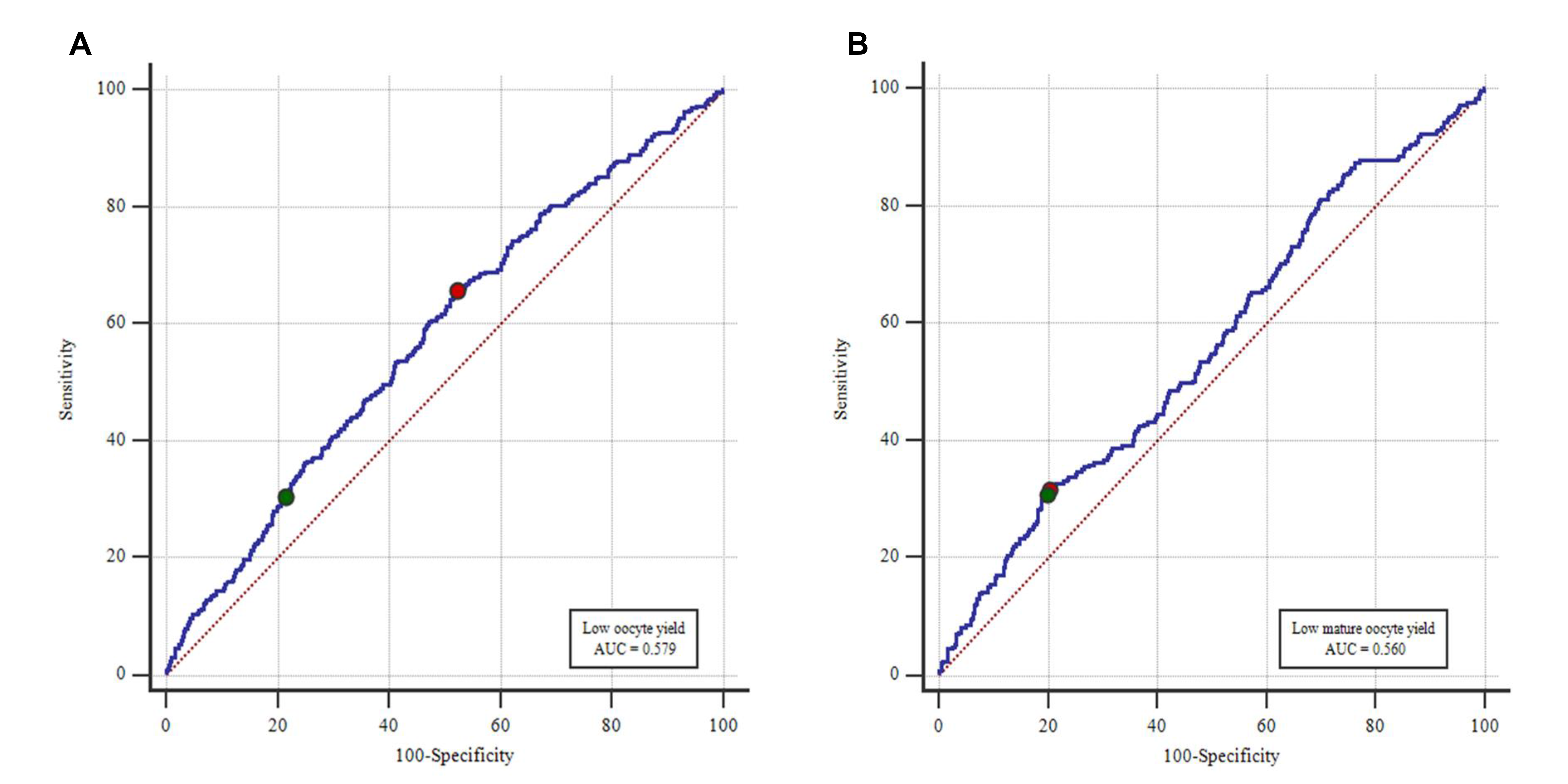

Supplement: Supplementary Figure 1 — Receiver operating characteristic curves of post-trigger E2 percentage increase for the prediction of (A) low oocyte yield and (B) low mature oocyte yield. The diagonal line is the reference line of no discrimination (area under the curve = 0.5). The marked red and green points correspond with the optimal cutoff value and the criterion value of 40%, respectively. E2, estradiol. [file Image_1.TIF]
