# Supplementary material for: A Higher Estradiol Rise After Dual Trigger in Progestin-Primed Ovarian Stimulation Is Associated With a Lower Oocyte and Mature Oocyte Yield in Normal Responders
Source: Front Endocrinol (Lausanne). 2019 Oct 9;10:696. doi: 10.3389/fendo.2019.00696 (PMC6794366; doi:10.3389/fendo.2019.00696)
Supplement: Supplementary Table 1 — Pregnancy outcomes of frozen-thawed embryos originating from groups stratified by the magnitude of E2 increase after dual trigger. [file Table_1.DOCX]

**Supplementary Table 1.** Pregnancy outcomes of frozen-thawed embryos originating from groups stratified by the magnitude of E_2_ increase after dual trigger

|  | <10.0% | 10.0–19.9% | 20.0–29.9% | 30.0–39.9% | ≥40.0% | *P* value for  difference | Adjusted *P* value for difference ^a^ |
| --- | --- | --- | --- | --- | --- | --- | --- |
| No. of patients | 257 | 331 | 329 | 261 | 300 |  |  |
| No. of FET cycles | 338 | 442 | 423 | 333 | 406 |  |  |
| No. of embryos transferred |  |  |  |  |  |  |  |
| Single | 73 (21.6) | 92 (20.8) | 79 (18.7) | 68 (20.4) | 92 (22.7) | 0.704 | - |
| Double | 265 (78.4) | 350 (79.2) | 344 (81.3) | 265 (79.6) | 314 (77.3) |  |  |
| Embryo stage at transfer, *n* (%) |  |  |  |  |  |  |  |
| Cleavage stage | 285 (84.3) | 372 (84.2) | 365 (86.3) | 290 (87.1) | 350 (86.2) | 0.723 | - |
| Blastocyst stage | 53 (15.7) | 70 (15.8) | 58 (13.7) | 43 (12.9) | 56 (13.8) |  |  |
| Endometrial preparation, *n* (%) |  |  |  |  |  |  |  |
| Natural cycle | 148 (43.8) | 186 (42.1) | 179 (42.3) | 132 (39.6) | 151 (37.2) | 0.452 | - |
| Mild stimulation | 108 (32.0) | 153 (34.6) | 152 (35.9) | 132 (39.6) | 156 (38.4) |  |  |
| Hormone replacement therapy | 82 (24.3) | 103 (23.3) | 92 (21.7) | 69 (20.7) | 99 (24.4) |  |  |
| Endometrial thickness (mm) | 10.10±1.91 | 10.08±2.12 | 10.25±2.02 | 10.23±2.06 | 10.28±2.25 | 0.683 | - |
| Pregnancy outcomes per transfer, *n*/*N* (%) |  |  |  |  |  |  |  |
| Implantation | 193/603 (32.0) | 289/792 (36.2) | 291/767 (37.9) | 220/598 (36.8) | 285/720 (39.6) | 0.066 | - |
| Positive pregnancy test | 166/338 (49.1) | 252/442 (57.0) | 239/423 (56.5) | 185/333 (55.6) | 240/406 (59.1) | 0.082 | 0.264 |
| Clinical pregnancy | 154/338 (45.6) | 225/442 (50.9) | 219/423 (51.8) | 173/333 (52.0) | 226/406 (55.7) | 0.104 | 0.458 |
| Ongoing pregnancy | 143/338 (42.3) | 199/442 (45.0) | 204/423 (48.2) | 161/333 (48.3) | 205/406 (50.5) | 0.187 | 0.753 |
| Pregnancy loss | 38/166 (22.9) | 72/252 (28.6) | 52/239 (21.8) | 32/185 (17.3) | 53/240 (22.1) | 0.086 | 0.128 |
| Live birth | 128/338 (37.9) | 180/442 (40.7) | 187/423 (44.2) | 153/333 (45.9) | 187/406 (46.1) | 0.110 | 0.551 |

Values are presented as mean ± SD or number (percentage). E_2_, estradiol; FET, frozen embryo transfer.

^a^ Analysis was adjusted for patient age, body mass index, infertility type, duration and diagnosis, additional infertility diagnosis (including polycystic ovarian syndrome and diminished ovarian reserve), number of embryos transferred, embryo stage at transfer, endometrial preparation and endometrial thickness.
